# Effects of endoscopic injection sclerotherapy for esophagogastric varices on portal hemodynamics and liver function

**DOI:** 10.1186/s12876-022-02422-7

**Published:** 2022-07-21

**Authors:** Ryuta Shigefuku, Hideaki Takahashi, Tsunamasa Watanabe, Nobuhiro Hattori, Hiroki Ikeda, Kotaro Matsunaga, Takuya Ehira, Tatsuya Suzuki, Nobuyuki Matsumoto, Chiaki Okuse, Motoh Iwasa, Hayato Nakagawa, Fumio Itoh, Michihiro Suzuki

**Affiliations:** 1 Department of Gastroenterology and Hepatology, Mie University Graduate School of Medicine, 2-174 Edobashi, Tsu, 514-8507 Japan; 2 Department of Gastroenterology and Hepatology, Yokohama City Seibu Hospital, St. Marianna University, Yokohama, Japan; 3 Division of Gastroenterology and Hepatology, Department of Internal Medicine, School of Medicine, St. Marianna University, Kawasaki, 216-8511 Japan; 4 Division of Gastroenterology and Hepatology, Department of Internal Medicine, Kawasaki Municipal Tama Hospital, Kawasaki, Japan; 5 Division of General Internal Medicine, Department of Internal Medicine, Kawasaki Municipal Tama Hospital, Kawasaki, Japan

**Keywords:** Endoscopic injection sclerotherapy, Indocyanine green retention at 15 min, Liver function, Portal hemodynamic, Portal hypertension

## Abstract

**Objectives:**

To identify patients suitable for endoscopic injection sclerotherapy (EIS) by evaluating their portal hemodynamics and liver function.

**Methods:**

We selected 58 patients with esophagogastric varices (EGV) and liver cirrhosis (LC) related to either hepatitis C virus (C) (n = 19), hepatitis B virus (n = 2), alcohol (AL) (n = 20), C + AL (n = 6), non-alcoholic steatohepatitis (n = 6), others (n = 3), or non-LC (n = 2). All patients underwent EIS. We measured their portal venous tissue blood flow (PVTBF) and hepatic arterial tissue blood flow (HATBF) using xenon computed tomography before and after EIS. We classified them into increased group and decreased group according to the PVTBF to identify the predictors that contribute to PVTBF increase post-EIS.

**Results:**

Low value of indocyanine green retention at 15 min (ICG-R_15_), the absence of paraesophageal veins, and low baseline PVTBF/HATBF (P/A) ratio predicted increased PVTBF in the multivariate logistic analysis (odds ratio (OR) 10.46, *p* = 0.0391; OR 12.45, *p* = 0.0088; OR 13.57, *p* = 0.0073). The protein synthetic ability improved 1 year post-EIS in increased group. Cox proportional hazards regression identified alcohol drinking (hazard ratio; 3.67, *p* = 0.0261) as an independent predictor of EGV recurrence.

**Conclusions:**

Patients with low ICG-R_15_, low P/A ratio, and the absence of paraesophageal veins were probable predictors of PVTBF improvement post-EIS. In addition, the improvement of hepatic hemodynamics likely enhanced liver function following EIS.

**Supplementary Information:**

The online version contains supplementary material available at 10.1186/s12876-022-02422-7.

## Introduction

Recently, endoscopic variceal ligation (EVL) has become increasingly popular as a safe and effective treatment for esophagogastric varices (EGV). Therefore, EVL is the first choice for esophageal variceal bleeding [1]. However, it tends to display lesser time to recurrent EGV, compared to endoscopic injection sclerotherapy (EIS). Therefore, EIS has been recommended in Japan to compensate for the disadvantages of EVL [2, 3]. The evaluation of hepatic hemodynamics before and after EIS is critical because it can predict systemic hemodynamics following changes in portal hemodynamics. However, researchers have not sufficiently elucidated changes in portal hemodynamics. Previous studies reported on changes in portal hemodynamics using Doppler ultrasonography (US) before and after EIS [4–6]. The portal tissue blood flow (PVTBF) using xenon computed tomography (Xe-CT) increases after the obliteration of EGV by EIS [7]. However, it is not always possible to increase PVBTF following the obliteration of varices. It is difficult to identify patients in advance who result in an increase in PVBTF post varices obliteration. In contrast, EIS is contraindicated for patients with refractory ascites, hypoalbuminemia, and uncontrollable encephalopathy. This is because hepatic failure supposedly occurs post-obliteration in such patients. Regardless of the hemodynamic change following the obliteration of EGV, the liver in some patients with cirrhosis cannot accept additional blood flow because of severe fibrosis, thereby preventing PVBTF increase. In this study, we aimed to identify patients suitable for EIS for EGV treatment, in terms of changes in their portal hemodynamics and liver function.

## Patients and methods

### Patients

**Fig. 1:**
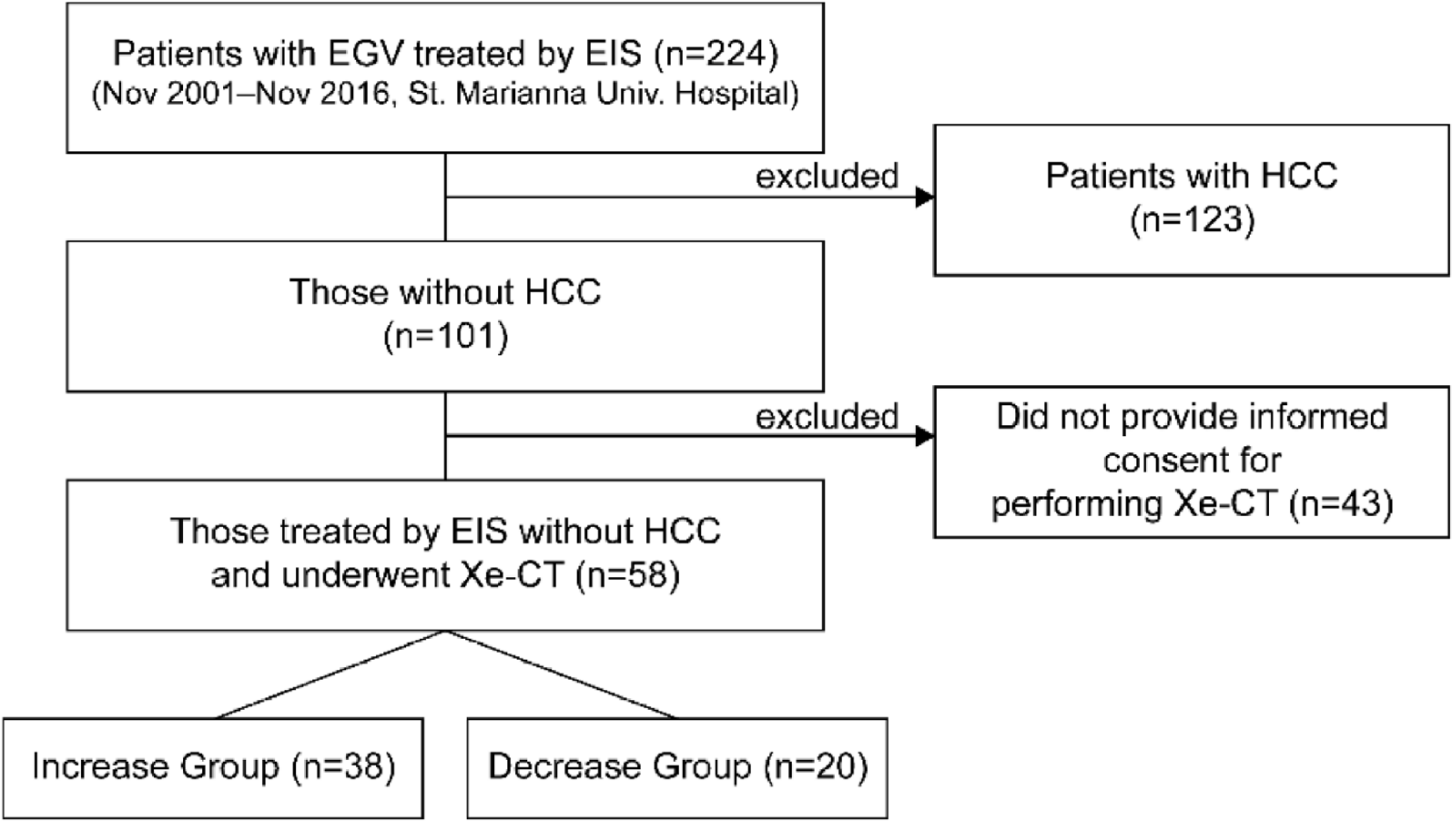
Flowchart for patient selection. Between November 2001 and November 2016, 224 patients with EGV were treated with EIS. We excluded 123 patients with hepatocellular carcinoma. Eventually, 58 patients who provided written informed consent, were enrolled in this study. We divided patients into two groups, namely increased group (IG) and decreased group (DG) following endoscopic sclerotherapy

Between November 2001 and November 2016, 224 patients with EGV were treated with EIS at St. Marianna university school of medicine hospital. Of these patients, 123 patients with hepatocellular carcinoma were excluded. Eventually, we enrolled 58 patients (38 men, 20 women; median age 65.5 years (range 36–78 years) who provided written informed consent (Fig. 1). EGV included esophageal varices (EV) alone and EV connecting with gastric varices. In addition, all patients with gastrorenal shunts were performed balloon occluded retrograde transvenous obliteration before EIS.

**Fig. 2:**
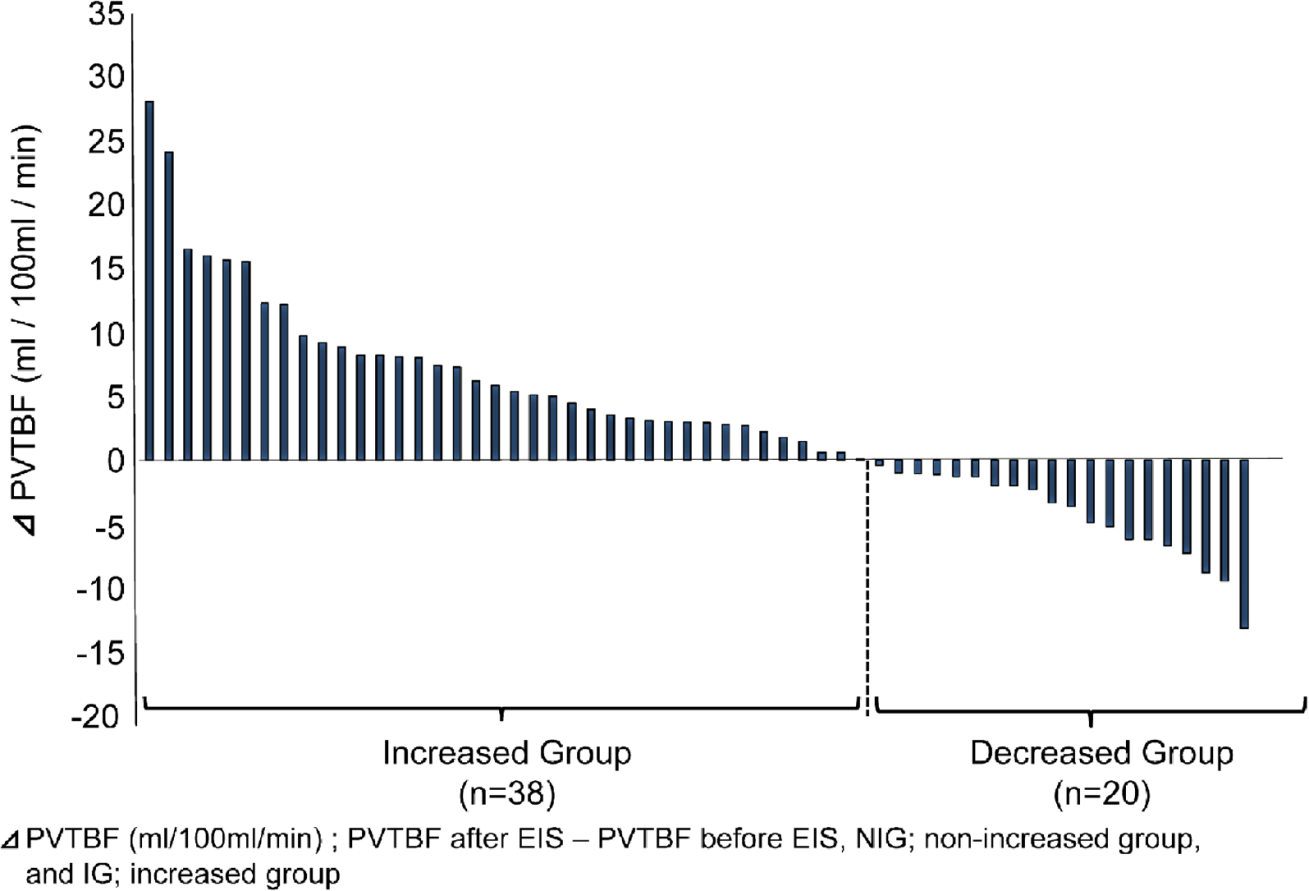
Definition for increased and decreased groups. All patients have been divided into two groups, namely increased (IG) and decreased groups (DG) after endoscopic sclerotherapy

All patients underwent PVTBF measurement using Xe-CT before and after EIS for EGV. While the increased group (IG) was defined as increased PVTBF after EIS, the decreased group (DG) was defined as decreased PVTBF following EIS. All patients were divided into the two groups, namely IG (n = 38) and DG (n = 20) (Fig. 2). We enrolled patients with liver cirrhosis (LC) related to either hepatitis C virus (C) (n = 19), hepatitis B virus (B) (n = 2), alcohol (AL) (n = 20), C + AL (n = 6), non-alcoholic steatohepatitis (n = 6), others (n = 3), or non-LC (n = 2). “Alcohol drinking” were defined as patients who continued to drink ethanol > 60 g/day before and after EIS. After confirming the above-mentioned criteria and the absence of hepatocellular carcinoma, we performed Xe-CT before and after EIS. All patients did not receive the recently initiated branched chain amino acids preparation, interferon, direct-acting antivirals, nucleoside analog, adrenocortical steroid, and ursodeoxycholic acid 1 year before and after EIS. Recurrent cases of EGV were defined as patients who demonstrated EGV bleeding or EGV with a therapeutic indication post-EIS. All study protocols were conducted in accordance with the ethics guidelines of the 1975 Declaration of Helsinki and were approved by the ethics committee at St. Marianna university school of medicine (Approval No. 480). This prospective study was performed from 2001 to 2018.

### Biochemical laboratory tests

Biochemical laboratory tests included the following parameters: albumin (Alb) (g/dL), prothrombin time (PT) (%), total bilirubin (T.B) (mg/dL), ammonia (NH_3_) (μg/dL) and indocyanine green retention at 15 min (ICG R_15_) (%). Serum Alb, T.B, PT and NH_3_ were measured using Iatrofine albumin (LSI Medience Corporation, Tokyo), IatroLQ (LSI Medience Corporation, Tokyo), Thromborel® S (Siemens, Tokyo) and Dimension® Clinical Chemistry System (Siemens, Tokyo), respectively. We evaluated chronological changes in serum albumin, prothrombin time, total bilirubin, NH_3_, Child–Pugh score, Model for end-stage liver disease (MELD) score and ICG-R_15_ before and after EIS.

### Definition of portosystemic shunts

The para-esophageal veins (Para-V) denotes azygos veins and enter the superior vena cava. The para-umbilical vein connects from the portal vein to systemic circulation. The gastrorenal shunt connects from the left gastric vein or from portogastric, short gastric veins to the left renal vein (Additional file 1: Fig. S1). We evaluated portosystemic shunts using enhanced CT.

### Xe-CT theory and imaging protocol

Xe is an inert gas present in the atmosphere in trace amounts. A high atomic weight and X-ray mass absorption coefficient facilitate the measurement of changes in tissue concentrations of Xe with CT. In Xe-CT, changes in Xe concentrations are measured over time in the hepatic tissue and spleen. By applying the Fick principle, a single blood supply model (inflow: arterial only, outflow: venous) can be fitted to a dual blood supply model (inflow: arterial and portal venous) to separately determine HATBF (ml/100 ml/min) and PVTBF (ml/100 ml/min) (Additional file 2: Fig. S2).

The imaging devices, protocol, and processing were the similar to that reported previously [8]. We used 25% stable Xe gas in conjunction with an AZ-726 Xe gas inhalation system (Anzai Medical, Tokyo, Japan). The wash-in and wash-out periods were of 4 min. The entire liver was CT-scanned at 1-min intervals at four levels, including the porta hepatis (nine scans in total, including the baseline scan) (Additional file 3: Fig. S3). Using an AZ-7000 W image processing system (Anzai Medical, Tokyo, Japan), we calculated the PVTBF, HATBF, and total hepatic tissue blood flow (THTBF), as the sum of PVTBF and HATBF, followed by the creation of maps. We used an Aquilion CT scanner (Toshiba Medical Systems, Tokyo, Japan), with exposure factors of 120 kV, 150 mA, and 13.8 mGy. The regions of interest (ROI) were selected at four levels, and areas with low reliability were automatically excluded. The mean of the four levels was used to determine the PVTBF and HATBF in each patient. THTBF was calculated as the sum of PVTBF and HATBF. Xe-CT was performed before and a week after EIS.

### Application and technique of EIS

All patients were performed as primary prophylaxis for variceal bleeding. The inclusion criteria of EIS were as follows: (i) EGV evaluated as F2 (nodular, moderately enlarged) or F3 (markedly enlarged) or red color signs on endoscopy, according to the classification system of the Japanese Society for Portal Hypertension and Esophageal Varices [9], (ii) > 20 years old, (iii) Child–Pugh classification of grade A or B, and (iv) the absence of refractory ascites. Ethanolamine oleate (5%EO, OLDAMIN®, ASKA Pharmaceutical. Co., Ltd., Tokyo, Japan) is the most commonly used sclerosant in Japan. We used a standard injection sclerotherapy technique. The sclerosant, 5% EO, was injected using an intravariceal technique. A maximum total volume of 0.4 mL/kg of 5% EO was injected at any one endoscopic procedure for the prevention of variceal bleeding. The endoscopic procedure was performed once a week until the varices were eradicated (Additional file 4: Fig. S4). In this study, argon plasma coagulation were not performed during the same hospitalization.

### Study design

We conducted a retrospective study aimed to clarify the independent predictors of decreased PVTBF after EIS using a multivariate logistic regression analysis (UMIN000008078). We evaluated chronological changes in biochemical laboratory tests before and after EIS (one year) and assessed the predictors of esophageal varices recurrence and mortality (5 years).

### Statistical analyses

Each parameter is expressed as median (first quartile-third quartile). We compared the patient characteristics between IG and the DG using the Mann–Whitney *U*-test. The paired-*t* test was performed to examine chronological changes in liver function tests before and after EIS. For each continuous variable, we selected the optimal cut-off value that maximized the sum of sensitivity and specificity using a receiver operating characteristic (ROC) analysis. A logistic regression analysis was performed for the multivariate analysis to evaluate the relationship between decreased PVTBF after EIS and clinical data. A cox proportional hazards regression analysis was conducted for the multivariate analysis to evaluate the relationship between esophageal varices recurrence and clinical data. Only variables deemed to be significant (*p* < 0.1) in the univariate analysis were included in the subsequent multivariate analysis. Statistical analyses were performed using JMP software program (SAS Institute, Cary, NC, USA) for the univariate and multivariate logistic regression analysis. Differences were considered significant at *p* < 0.05.

## Results

### Patient characteristics

The DG demonstrated a higher proportion of older patients, women, and significantly higher ICG-R_15_, PVTBF, and P/A ratio. There were no significant differences in the serum Alb, T.B, PT, NH_3_, number of EIS sessions, total volume of sclerosant, Child–Pugh class, HATBF, and THTBF. Furthermore, we found significantly higher Para-V in the DG (Table 1).

**Table 1:** Characteristics of patients

	All (n = 58)	Increased Group (n = 38)	Decreased group (n = 20)	p^*^
Age (years)	65.5 (54.5–71.0)	60.0 (52.0–68.0)	69.5 (65.0–75.0)	**0.0023**
Gender (male/female)	38/20	29/9	9/11	**0.0478**
*Etiology of liver disease*				
HCV-LC	19	9	10	0.0511
HBV-LC	2	2	0	–
Alcohol-LC	20	16	4	0.0954
HCV + Alcohol-LC	6	4	2	0.9630
NASH (NBNC)-LC	6	4	2	0.9630
PBC-LC	1	0	1	–
AIH-LC	2	2	0	–
Non-cirrhosis	2	1	1	0.6600
*Liver function*				
AST (U/L)	42.0 (34.0–49.0)	37.0 (31.0–49.0)	44.5 (35.3–48.0)	0.4007
ALT (U/L)	26.0 (17.0–36.0)	24.0 (16.0–36.5)	26.5 (21.3–30.8)	0.7242
Platelet count (× 10^4^/μL)	8.1 (5.6–10.7)	7.8 (5.8–10.2)	9.6 (5.5–12.0)	0.3560
Albumin (g/dL)	3.4 (3.2–4.0)	3.6 (3.3–4.1)	3.3 (3.1–3.5)	0.0512
Total Bilirubin (mg/dL)	1.0 (0.8–1.3)	0.9 (0.8–1.0)	1.1 (0.9–2.0)	0.1206
Prothrombin time (%)	70.0 (65.0–77.3)	70.0 (65.5–74.0)	71.2 (65.0–78.0)	0.7151
Creatinine (mg/dL)	0.72 (0.65–0.88)	0.78 (0.69–0.93)	0.71 (0.62–0.81)	0.1666
Ammonia (μg/dL)	61.0 (49.0–76.0)	64.5 (51.0–73.8)	59.0 (49.0–119.0)	0.7755
ICG-R_15_ (%)	30.0 (20.0–37.0)	27.0 (18.5–33.0)	36.0 (26.5–42.0)	**0.0170**
FIB4 index	5.87 (4.66–9.11)	6.18 (4.72–9.00)	5.62 (4.76–10.62)	0.9256
Child–Pugh (CP) class (A /B) (CP-A;%)	35/21 (60.3)	26/11 (70.3)	9/10 (47.4)	0.1113
Child–Pugh (CP) score (5/6/7/8/9)	19/15/13 /7/2	14/11/7/5/0	5/4/6/2/2	–
MELD score	14.9 (13.5–16.2)	15.1 (13.9–15.9)	14.6 (13.5–17.0)	0.7542
*Endoscopic findings*				
Form 1/2/3	5/37/16	2/23/13	3/14/3	–
Red color sign (+/-)	46/12	28/10	18/2	0.3122
Number of EIS sessions (times)	2.0 (1.0–2.0)	2.0 (1.0–2.0)	1.5 (1.0–2.0)	0.8875
Total volume of sclerosant (ml)	15.0 (11.0–20.0)	16.5 (12.0–20.0)	14.0 (10.0–16.0)	0.0656
*Complications of portal hypertension*				
Paraesophageal veins (Para-V) (+/-)	22/36	10/28	12/8	**0.0168**
Paraumbilical veins (+/-)	21/37	13/25	8/12	0.6827
Gastrorenal shunts (after B-RTO) (+/-)	6/52	4/34	2/18	0.7395
Splenomegaly (over 11 cm) (+/-)**	37/21	23/15	14/6	0.5518
Ascites (+/-)	12/46	9/29	3/17	0.4527
*Liver hemodynamics*				
PVTBF (ml/100 ml/min)	27.1 (22.7–31.2)	25.5 (21.4–28.6)	29.7 (25.3–37.2)	**0.0151**
HATBF (ml/100 ml/min)	19.4 (14.8–25.1)	19.4 (15.7–25.1)	18.5 (11.7–24.2)	0.2695
THTBF (ml/100 ml/min)	47.5 (39.9–60.7)	45.7 (39.9–54.5)	49.9 (56.3–65.5)	0.3304
P/A ratio	1.4 (1.0–1.9)	1.3 (1.0–1.6)	1.7 (1.1–2.0)	**0.0073**

All data are expressed as median (first quartile-third quartile)

*Mann–Whitney-*U* test (Increased Group vs. Decreased group)

**The maximum diameter of spleen length by enhanced computed tomography

^‡^*IG*, increased group; *DG*, decreased group; *NASH*, nonalcoholic steatohepatitis; *NBNC*, non-B non-C; *PBC*, primary biliary cholangitis; *AIH*, autoimmune hepatitis; *AST*, aspartate aminotransferase; *ALT*, alanine aminotransferase, *ICG-R*_*15*_, indocyanine green retention at 15 min; *FIB4 index*, fibrosis-4; *MELD*, Model for End-Stage Liver Disease; *EIS*, endoscopic injection sclerotherapy; *B-RTO*, balloon-occluded retrograde transvenous obliteration; *PVTBF*, portal venous tissue blood flow; *HATBF*, hepatic arterial tissue blood flow; *THTBF*, total hepatic tissue blood flow; and *P/A ratio*, PVTBF/HATBF ratio

### Distinguishing IG from DG using baseline ICG-R_15_ and P/A ratio

We attempted to distinguish IG and DG using baseline ICG-R_15_ and P/A ratio. ICG-R_15_ and P/A ratio before EIS displayed significantly lower values in the IG than those in the DG. In other words, the ROC curve and the area under the ROC (AUC) using the P/A ratio and ICG-R_15_ could predict definitive DG. The AUC value of ICG-R_15_ and P/A ratio were 0.70 and 0.71, respectively. A cut-off ICG-R_15_ value of 30% predicted DG with a specificity and sensitivity of 67% and 71%, respectively. A cut-off P/A ratio of 1.5 predicted DG with a specificity and a sensitivity of 68% and 65%, respectively.

### Independent predictors of increased PVTBF after EIS

Gender, age, low baseline ICG-R_15_ (< 30%), the absence of Para-V, and low baseline P/A ratio (< 1.5) were predictors of increased PVTBF after EIS by the univariate logistic regression analysis. The multivariate logistic regression analysis identified high baseline ICG-R_15_ (< 30%) (odds ratio (OR) 10.46, *p* = 0.0391), the absence of Para-V (OR 12.45, *p* = 0.0088), and low baseline P/A ratio (OR 13.57, *p* = 0.0073) as independent predictors of increased PVTBF post-EIS (Table 2).

**Table 2:** Univariate and multivariate logistic regression analysis for predictors that increase portal blood flow following endoscopic injection sclerotherapy

Factors	Univariate analysis			Multivariate analysis		
	OR	95% CI	*p*	OR	95% CI	*p*
Gender (male)	0.25	0.0770 to 0.7916	0.0180	0.23	0.0186 to 1.9875	0.1820
Age (under 65 year-old)	4.60	1.4521 to 16.6612	0.0088	6.25	0.9121 to 62.0948	0.0784
Child–Pugh (under 7)	2.31	0.7490 to 7.3662	0.1447	–	–	–
ICG-R_15_ (under 30%)	4.35	1.1917 to 18.7385	0.0255	10.46	1.4460 to 152.5174	**0.0391**
Para-V	4.20	1.3616 to 13.8270	0.0123	12.45	1.7990 to 170.3335	**0.0088**
P/A ratio (under 1.5)	4.02	1.3156 to 13.2760	0.0143	13.57	1.9267 to 192.6336	**0.0073**

*OR*, odds ratio; *95% CI*, 95% confidence interval; *ICG-R*_*15*_, indocyanine green retention at 15 min; *Para-V*, paraesophageal veins; and *P/A ratio*, portal venous tissue blood flow/hepatic arterial tissue blood flow ratio

### Chronological changes in liver function test values before and after EIS

We evaluated chronological changes in the serum Alb, PT, T.B, NH_3_, Child–Pugh score, MELD score and ICG-R_15_ before and after EIS. Serum Alb at 12 months post-EIS improved in the IG (*p* = 0.0036). The PT at 1 month and 12 months after EIS improved in the IG (p = 0.0234, p = 0.0333, respectively). T.B at 1 week after EIS improved in the IG (*p* = 0.0084). NH_3_ in 1 week, 1 month, and 6 month following EIS improved (*p* = 0.0045, *p* = 0.0209, *p* = 0.0075, respectively) in the IG. Child–Pugh score at 12 months post-EIS improved in the IG (p = 0.0475). MELD score at 1 week and 12 months post-EIS improved in the IG (*p* = 0.0215, *p* = 0.0321, respectively). ICG-R_15_ at 1 week post-EIS improved in the IG (*p* = 0.0024). Furthermore, the protein synthetic ability also improved in the IG (Table 3).

**Table 3:** Chronological changes in liver function tests before and after endoscopic injection sclerotherapy

Parameter		Before EIS	1 W after EIS	P^*^	1 M after EIS	p^*^	6 M after EIS	p^*^	12 M after EIS	p^*^
Albumin (g/dL)	IG	3.60 (3.30–4.10)	3.60 (3.30–4.00)	0.8993	3.70 (3.50–4.00)	0.3287	3.85 (3.63–4.10)	0.1165	3.90 (3.68–4.13)	**0.0036**
	DG	3.30 (3.10–3.50)	3.35 (3.15–3.55)	0.9471	3.60 (3.13–3.83)	0.1715	3.40 (3.00–3.90)	0.8317	3.60 (3.05–3.70)	0.4347
PT (%)	IG	70.0 (65.5–74.0)	71.0 (66.0–76.0)	0.2148	69.0 (64.5–79.0)	**0.0234**	74.0 (66.0–85.0)	0.0907	74.0 (67.0–79.0)	**0.0333**
	DG	71.0 (65.0–78.0)	67.5 (65.0–78.0)	0.1243	73.5 (66.8–81.8)	0.5898	69.5 (62.3–80.3)	0.8867	63.0 (56.0–84.5)	0.7315
Tbil (mg/dL)	IG	0.9 (0.8–1.0)	0.8 (0.6–1.0)	**0.0084**	1.0 (0.7–0.4)	0.4970	0.9 (0.7–1.2)	0.9418	1.1 (0.8–1.5)	0.8687
	DG	1.1 (0.9–2.0)	0.9 (0.7–1.8)	0.6021	1.2 (0.8–2.4)	0.4895	1.2 (0.9–1.8)	0.4725	1.3 (1.0–1.8)	0.4885
NH_3_ (μg/dL)	IG	64.5 (51.0–73.8)	32.0 (18.0–42.0)	**0.0045**	46.0 (37.5–61.0)	**0.0209**	43.0 (37.5–61.0)	**0.0075**	51.0 (37.5–64.0)	0.1133
	DG	59.0 (49.0–118.0)	46.0 (33.0–56.0)	0.0778	36.0 (24.0–78.0)	0.2062	92.0 (80.0–115.5)	0.8486	64.0 (31.5–92.8)	0.3215
Child–Pugh score	IG	6.00 (5.00–6.00)	6.00 (5.00–6.00)	0.3332	6.00 (5.00–6.00)	0.3343	5.50 (5.00–6.00)	0.1110	5.00 (5.00–5.75)	**0.0475**
	DG	7.00 (6.00–7.00)	6.50 (6.00–7.00)	0.6305	5.50 (5.00–7.00)	0.2360	6.50 (5.00–7.00)	0.6776	7.00 (5.00–7.00)	1.000
MELD score	IG	15.1 (13.9–15.9)	14.9 (13.5–15.8)	**0.0215**	15.0 (13.5–15.9)	0.3572	14.7 (13.2–15.7)	0.1025	14.7 (13.6–16.0)	**0.0321**
	DG	14.6 (13.5–17.0)	14.2 (13.4–15.9)	0.8705	14.8 (13.5–17.2)	0.8830	14.8 (13.3–16.6)	0.8574	14.4 (13.0–15.3)	0.9077
ICG-R_15_ (%)	IG	27.0 (18.5–33.0)	22.5 (16.0–28.8)	**0.0024**	–	–	–	–	–	–
	DG	36.0 (26.5–42.0)	29.0 (25.0–41.0)	0.2711	–	–	–	–	–	–

All data are expressed as median (first quartile-third quartile)

*Before EIS vs 1 W, 1 M, 6 M, 12 M (paired-*t* test)

^‡^*EIS*, endoscopic injection sclerotherapy; *W*, week; *M*, months; *PT*, prothrombin time; *Tbil*, total bilirubin; *NH*_*3*_, ammonia; *MELD*, Model for End-Stage Liver Disease; *ICG-R*_*15*_, indocyanine green retention at 15 min

IG, increased group; and DG, decreased group

### Univariate and multivariate cox proportional hazards regression analysis for predictors of esophageal varices recurrence

Using univariate and multivariate cox proportional hazards regression, alcohol drinking (hazard ratio; 3.67, p = 0.0261) were identified as independent predictors of EGV recurrence (Table 4).

**Table 4:** Univariate and multivariate Cox proportional hazards regression analysis for predictors of esophageal varices recurrence

Predictors	Univariate analysis			Multivariate analysis		
	HR	95% CI	*P* value	HR	95% CI	*P* value
Age	2.66	0.9309 to 8.6389	0.0681	0.75	0.2083 to 2.4578	0.6432
Gender (male)	0.53	0.1442 to 1.6018	0.2694	–	–	–
Child–Pugh score (over 7)	2.69	0.9043 to 8.0179	0.0742	2.58	0.8526 to 7.8115	0.0918
Alcohol drinking	4.10	1.4343 to 13.3535	0.0083	3.67	1.1638 to 13.0946	**0.0261**
DG	0.60	0.1633 to 1.7852	0.3669	–	–	–
Para-V	0.97	0.4566 to 1.7785	0.9634	–	–	–
Splenomegaly	0.90	0.3185 to 2.9195	0.8541	–	–	–
Ascites	2.14	0.6952 to 6.2393	0.1766	–	–	–

Bold indicates significcuntly difference (*p* < 0.05)

*HR*, hazard ratio; *95% CI*, 95% Confidence interval; *DG*, decreased group; and *Para-V*, para esophageal veins

### Logistic regression analysis for the recurrence rate of esophagogastric varices in each factor

**Fig. 3:**
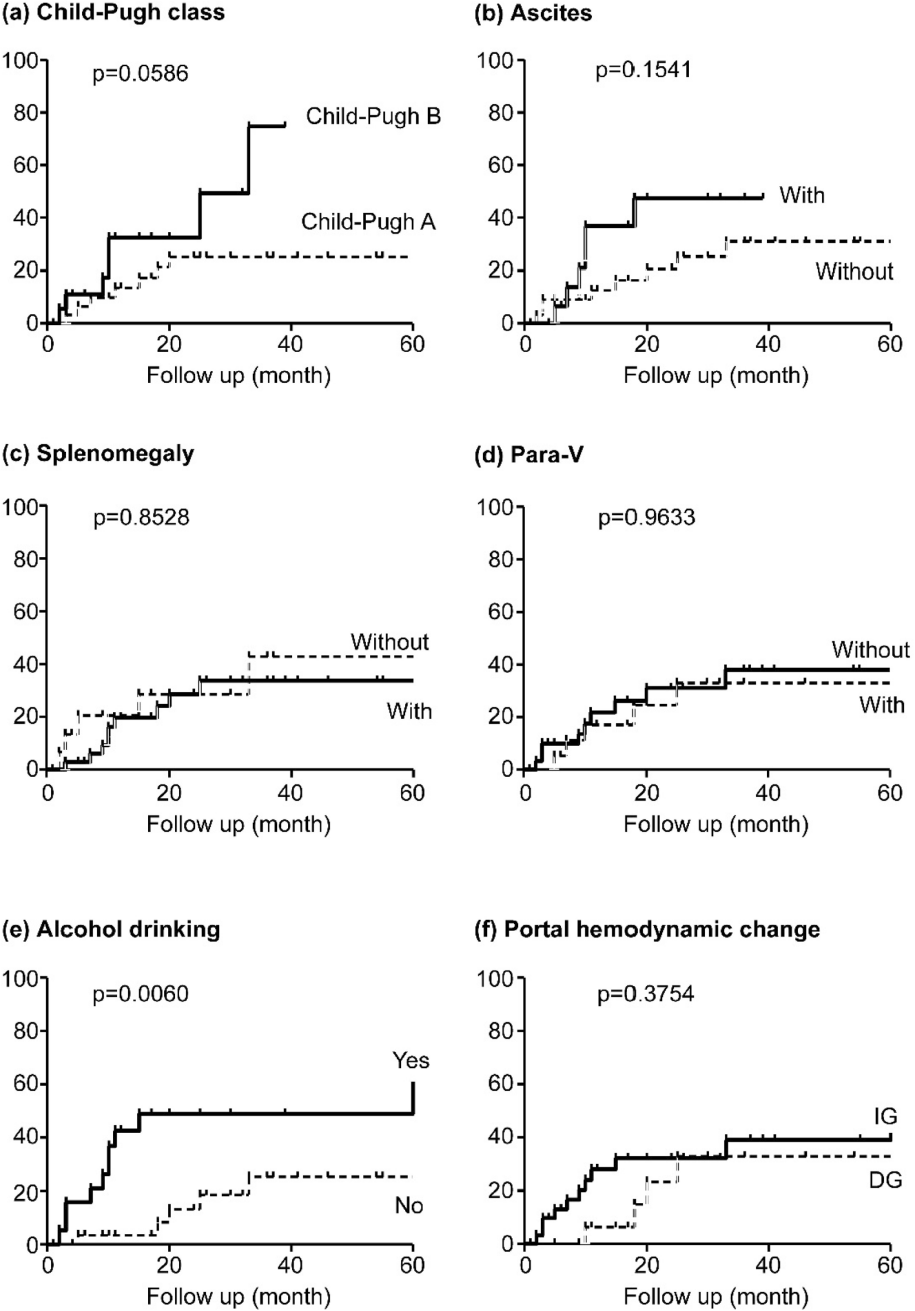
The recurrence rate of esophagogastric varices for each factor. Alcohol drinking display significantly higher recurrence rate of EGV. Patients with Child–Pugh score ≥ 7 tend to display high recurrence rate of EGV

Alcohol drinking demonstrated a significantly higher recurrence rate of EGV (P = 0.0060). Patients with high Child–Pugh score (> 7) tended to display a high recurrence rate of EGV (P = 0.0586). In contrast, patients with ascites, splenomegaly, and Para-V did not demonstrate a high recurrence rate. Portal hemodynamic change after EIS did not affect the recurrence rate of EGV (Fig. 3).

### Comparison of IG and DG mortality

There were 7 dead cases (18%) in IG and 8 cases (40%) in DG. We did not find the significant difference of on mortality between IG and DG (*p* = 0.1707, Log-rank test). In IG, 6 cases resulted in liver failure and 1 case eventually developed HCC. In DG, 3 cases resulted in liver failure, 3 cases eventually developed HCC and 2 cases resulted in death due to other causes.

## Discussion

EIS for EGV occludes not only EGV but also the feeding vein. An EIS-mediated increase in the portal flow likely improves liver function. However, there are few detailed reports on the relation between liver function and portal hemodynamics.

Before and after EIS, while some studies reported on no change in the hepatic blood flow (HBF), others reported on an increase in HBF. The increase in HBF before and after EIS improved hepatic synthesis ability [4, 5]. However, it is difficult to discriminate between cases with increased HBF post-EIS. Xe-CT is widely used in neurosurgical practice to evaluate the cerebral tissue blood flow [10]. We have previously evaluated HBF in chronic liver disease using Xe-CT (11–14). The portal flow increased after EIS in some cases; however, in some cases it did not increase after EIS. In this study, we elucidated the characteristics of cases without increased portal flow after EIS by evaluating the portal hemodynamics before and after EIS [7]. The PVTBF increased in 66% cases following EIS. According to Sugimoto’s report, the portal blood flow increased in 60% of the patients [5], consistent to our findings. Generally, portal blood flow should be increased after EIS [5]. However portal blood flow in some patients was decreased after EIS in this study. We speculate two reasons why PVTBF decreased after EIS. First, it is suggested that para-esophageal venous flow increased after EIS [22], followed by decrease of hepatic tissues blood flow as the result of it. Second, because many of the patients in DG tended to have poor liver function, it is suggested that hepatic sinusoidal pressure increased after EIS, followed by decrease of hepatic tissues blood flow as the result of it. Doppler US measures intravascular blood flow calculated by measuring flow velocities and vessel diameters of the portal vein and hepatic artery. In contrast, Xe-CT measures tissue blood flow. Tissue blood flow is similar to and different from intravascular blood flow. It is suggested that intravascular portal trunk blood flow increases after EIS by doppler US, in contrast portal venous tissue blood flow decreases by Xe-CT after EIS in some cases. That is, Xe-CT have greater potential to evaluate true hepatic tissue blood flow and hepatic hemodynamics in detail after EIS.

High value of ICG-R_15_, the presence of Para-V, and high P/A ratio were the independent predictors of decreased PVTBF after EIS by the multivariate analysis. ICG-R_15_ was reportedly a non-invasive marker of portal hypertension [15, 16] and EGV [17]. The diagnostic ICG-R_15_ cut-off value to detect esophageal varices was 22.9% [17]. Other report mentioned that the ICG-R_15_ cut-off value for predicting portal hypertension according to hepatic venous pressure gradient (HVPG) was 16.0%, with a sensitivity and specificity of 72.3% and 79.0%, respectively. The cut-off value for distinguishing IG from DG was 30%. Yamazaki et al. reported that the presence of splenomegaly and ICG-R_15_ > 30% were significant predictors of worsening EGV [18], thus supporting our results. Furthermore, we confirmed the improvement of liver function tests and ICG-R_15_ following EIS in the IG. According to Iwata et al., the total bile acid, ICG-R_15_, and 99mTc-GSA scintigraphy significantly improved after sclerotherapy [19]. This report supports our data because both ICG-R_15_ and PVTBF improved post-EIS in the present study. In addition, we confirmed increased portal flow increases and improved hepatic hemodynamics because of EGV obliteration.

The presence of Para-V was considered the route to reduce or decompress excessive portal pressure following EIS. Toyonaga et al. evaluated portal hemodynamics and transhepatic portal venographic findings before and after prophylactic sclerotherapy. The prevalence of extravariceal portosystemic shunts was greater in patients with decreased portal pressure than in those with increased portal pressure. Therefore, the presence of extravariceal portosystemic shunts may prevent further increases in the portal pressure [20]. Mizumoto et al. examined changes in portal hemodynamics by doppler US and percutaneous transhepatic portography after EIS, in relation to post-treatment relapse. In other words, the frequent relapse of varices results from the insufficient blockage of blood flow from the left gastric vein to the lower esophagus. However, patients with a patent Para-V demonstrated satisfactory long-term effects obtained by EVL-EIS combination therapy [21]. In contrast, GR shunt did not affect on hemodynamic change. There were 6 cases with GR shunt, and all cases (IG 4 cases, DG 2 cases) underwent B-RTO. In patients who were performed B-RTO, EIS was performed 1 month after B-RTO. There was no significant difference between in 6 patients with GR shunt and 52 patients without GR shunt PVTBF before EIS and ΔPVTBF (*p* = 0.6162, *p* = 0.6162, respectively), and it was considered that there was less affected by B-RTO on hemodynamics in this study.

Kassem et al. reported on an increase in the diameter of azygos veins (Para-V) and blood flow volume index using endoscopic ultrasonography following variceal obliteration [22]. Therefore, Para-V plays a role in relieving portal pressure elevation post-EIS.

The P/A ratio denotes portal hemodynamics in the liver. Low P/A ratio reveals a status, i.e., a decrease in portal venous blood flow and a compensatory increase in arterial blood flow with the progression to liver cirrhosis, also called “liver with hepatic arterial dominance.” The formation of EGV is one of factors that decrease PVTBF and lower the P/A ratio. Obliteration of EGV by EIS can increase the PVTBF and improve portal hemodynamics in patients with lower P/A ratio. Kako et al. reported that portosystemic shunts occlusion increased the portal venous blood flow and decreased the hepatic arterial blood flow, thereby improving the liver profile [23], thus supporting our findings.

Following EIS, the portal venous pressure increased when over 70% of the study subjects comprised patients with Child–Pugh B or C-LC [24]. The capacity of the sinusoid may also contribute to changes in PVTBF post-EIS. In Child–Pugh A or B-LC, some cases display increased or decreased PVTBF following EIS. Using the multivariate cox proportional hazards regression, DG was not identified a predictor of EGV recurrence. Therefore, changes in portal hemodynamics following EIS did not affect EGV recurrence in those with Child–Pugh A or B-LC. Alcohol drinking were identified as independent predictors of EGV recurrence and other report also supports our results [25]. On the other hand, although EIS can lead to the short-term (one year) improvement of liver function, the effect cannot lead to affecting the prognosis and the recurrence of EGV for a long term (5 years).

The portal venous pressure is likely to increase following EIS; however, our failure to measure the HVPG and changes in portal venous pressure is a limitation of this study. Furthermore, since the small number of cases is also a limitation of this study, it is hoped that the number of cases will be increased and verified by the same method in the future. Patients with increased PVTBF after EIS may not necessarily display excessive portal venous pressure owing to improved protein synthetic ability after EIS. In addition, portal hemodynamic change after EIS did not affect the recurrence rate of EGV. In particular, the obliteration of EGV will likely lead to increased PVTBF and improved portal hemodynamics in patients with lower P/A ratio.

## Conclusion

Patients with low ICG-R15, low P/A ratio, and the absence of Para-V were probable predictors of PVTBF improvement post-EIS. In addition, the improvement of hepatic hemodynamics likely enhanced liver function following EIS.

## Supplementary Information

**Additional file 1.** **Supplementary Figure 1.** Definition for portosystemic shunts The para-esophageal veins (Para-V) (arrows) denote azygos veins and enter the superior vena cava. The para-umbilical vein connects from the portal vein to systemic circulation. The gastrorenal shunt (arrow head) connects from the left gastric vein or portogastric, short gastric veins to the left renal vein.

**Additional file 2.** **Supplementary Figure 2.** Xe-CT theory By applying the Fick principle, a single blood supply model (inflow: arterial only, outflow: venous) can be fitted to a dual blood supply model (inflow: arterial and portal venous) to separately determine HATBF (ml/100 ml/min) and PVTBF (ml/100 ml/min).

**Additional file 3.** **Supplementary Figure 3.** Xe-CT protocol The wash-in and wash-out periods were of 4 min. The entire liver was CT-scanned at 1-min intervals at four levels, including the porta hepatis (nine scans in total, including the baseline scan).

**Additional file 4** **Supplementary Figure 4.** Endoscopic findings of esophageal varices before and after EIS (A) Before EIS (F2 esophageal varices), (B) After EIS (White cord). EIS has been performed until the varices were eradicated.

## Data Availability

The datasets generated and/or analysed during the current study are not publicly available due to contain some personal information, but are available from the corresponding author on reasonable request.

## Abbreviations

EGV: Esophagogastric varices
EIS: Endoscopic injection sclerotherapy
Xe-CT: Xenon computed tomography
HBF: Hepatic blood flow
HATBF: Hepatic arterial tissue blood flow
PVTBF: Portal venous tissue blood flow
THTBF: Total hepatic tissue blood flow
IG: Increased group
DG: Decreased group
CLD: Chronic liver disease
LC: Liver cirrhosis
B: Hepatitis B virus
C: Hepatitis C virus
AL: Alcohol
NASH: Non-alcoholic steatohepatitis
AIH: Autoimmune hepatitis
PBC: Primary biliary cholangitis
ICG-R_15_: Indocyanine green retention at 15 min
US: Ultrasonography
ROI: Region of interest
HVPG: Hepatic venous pressure gradient
