# Supplementary material for: Effects of endoscopic injection sclerotherapy for esophagogastric varices on portal hemodynamics and liver function
Source: BMC Gastroenterol. 2022 Jul 21;22:350. doi: 10.1186/s12876-022-02422-7 (PMC9306194; doi:10.1186/s12876-022-02422-7)
Supplement: Supplementary file 1 — Additional file 1. Supplementary Figure 1. Definition for portosystemic shunts The para-esophageal veins (Para-V) (arrows) denote azygos veins and enter the superior vena cava. The para-umbilical vein connects from the portal vein to systemic circulation. The gastrorenal shunt (arrow head) connects from the left gastric vein or portogastric, short gastric veins to the left renal vein. [file 12876_2022_2422_MOESM1_ESM.pptx]

## Slide 1
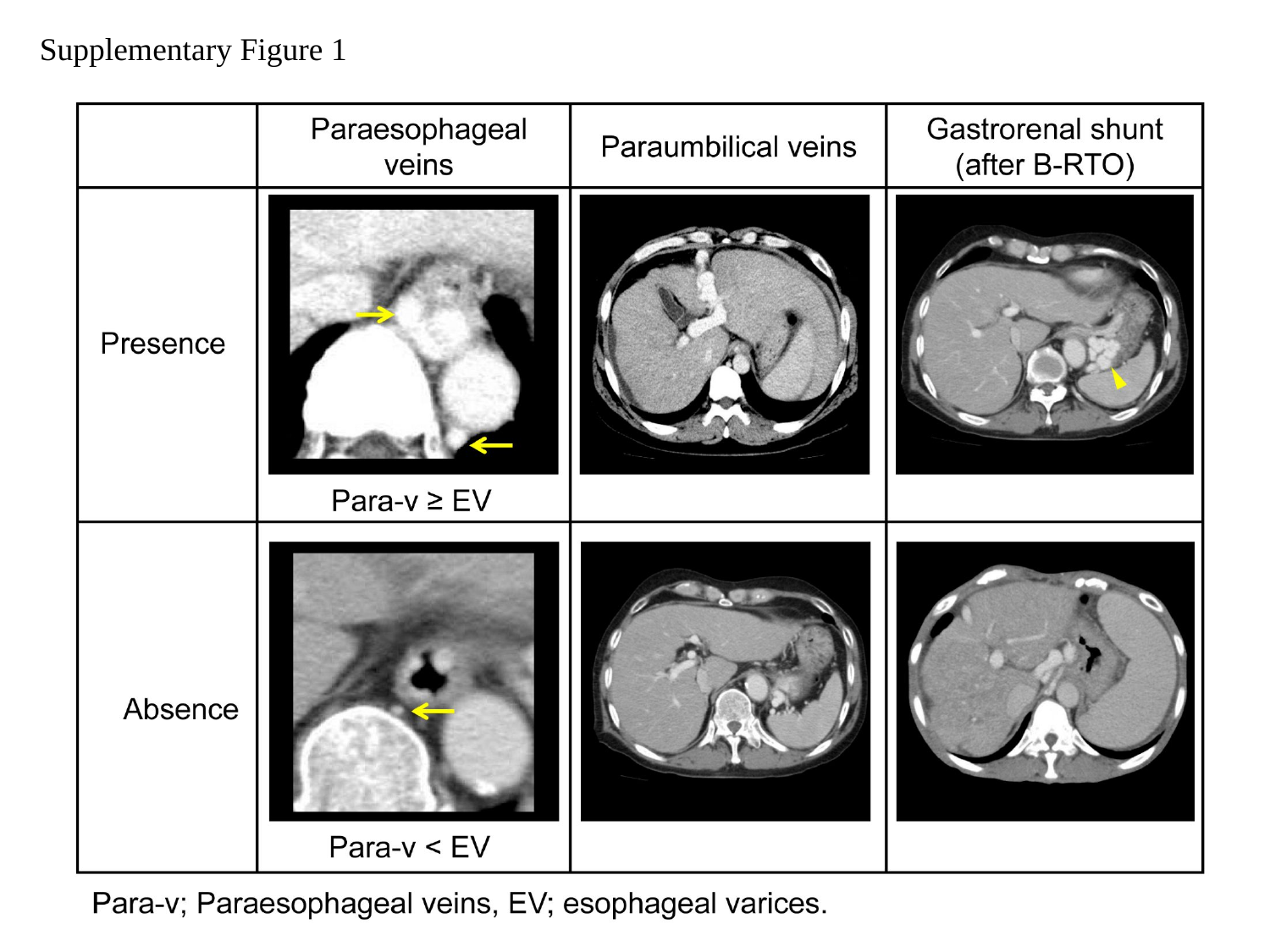

Supplementary Figure 1

## Slide 2
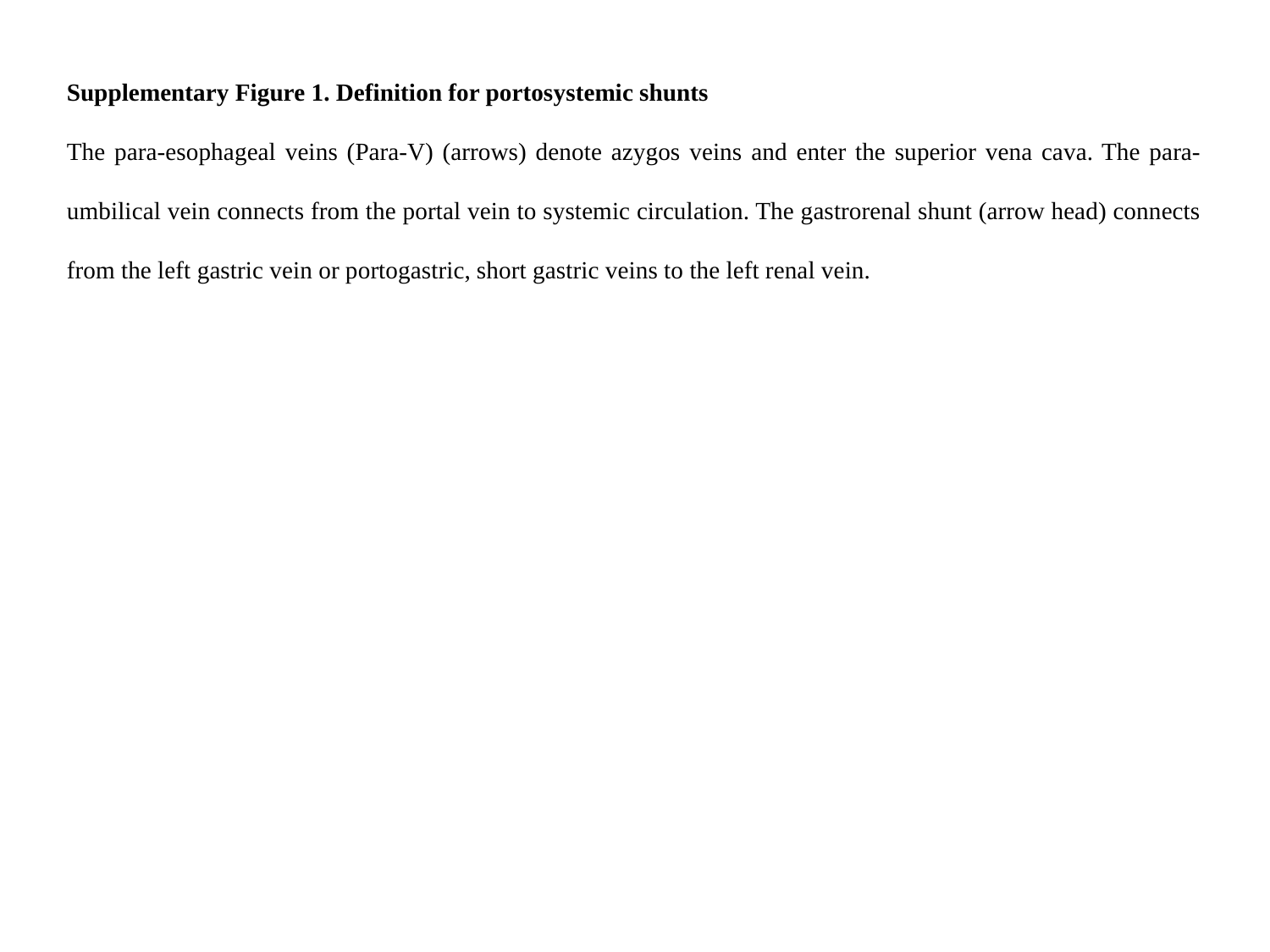

Supplementary Figure 1. Definition for portosystemic shunts
The para-esophageal veins (Para-V) (arrows) denote azygos veins and enter the superior vena cava. The para-umbilical vein connects from the portal vein to systemic circulation. The gastrorenal shunt (arrow head) connects from the left gastric vein or portogastric, short gastric veins to the left renal vein.
