# Supplementary material for: Effects of endoscopic injection sclerotherapy for esophagogastric varices on portal hemodynamics and liver function
Source: BMC Gastroenterol. 2022 Jul 21;22:350. doi: 10.1186/s12876-022-02422-7 (PMC9306194; doi:10.1186/s12876-022-02422-7)
Supplement: Supplementary file 2 — Additional file 2. Supplementary Figure 2. Xe-CT theory By applying the Fick principle, a single blood supply model (inflow: arterial only, outflow: venous) can be fitted to a dual blood supply model (inflow: arterial and portal venous) to separately determine HATBF (ml/100 ml/min) and PVTBF (ml/100 ml/min). [file 12876_2022_2422_MOESM2_ESM.pptx]

## Slide 1
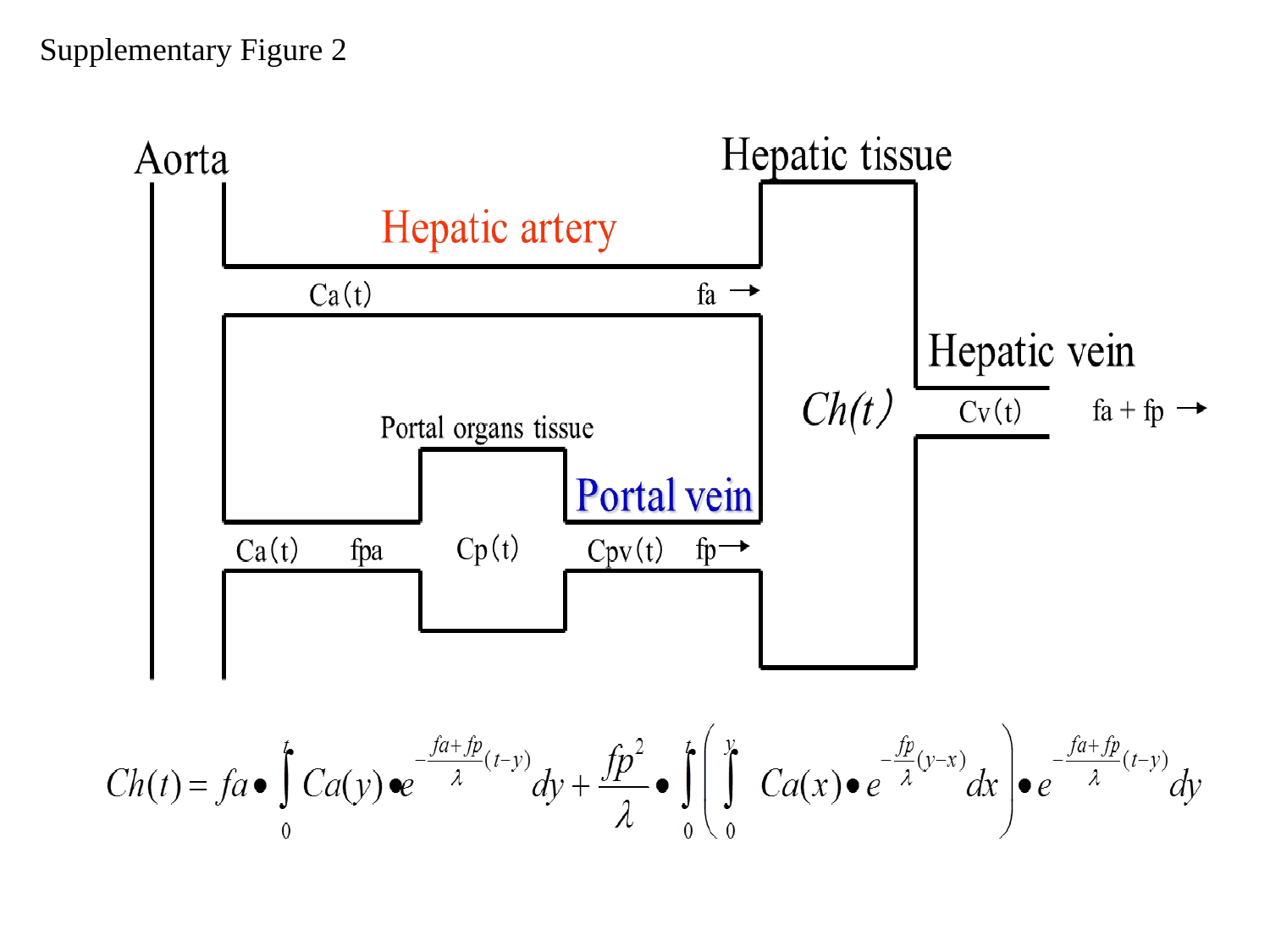

Supplementary Figure 2

## Slide 2
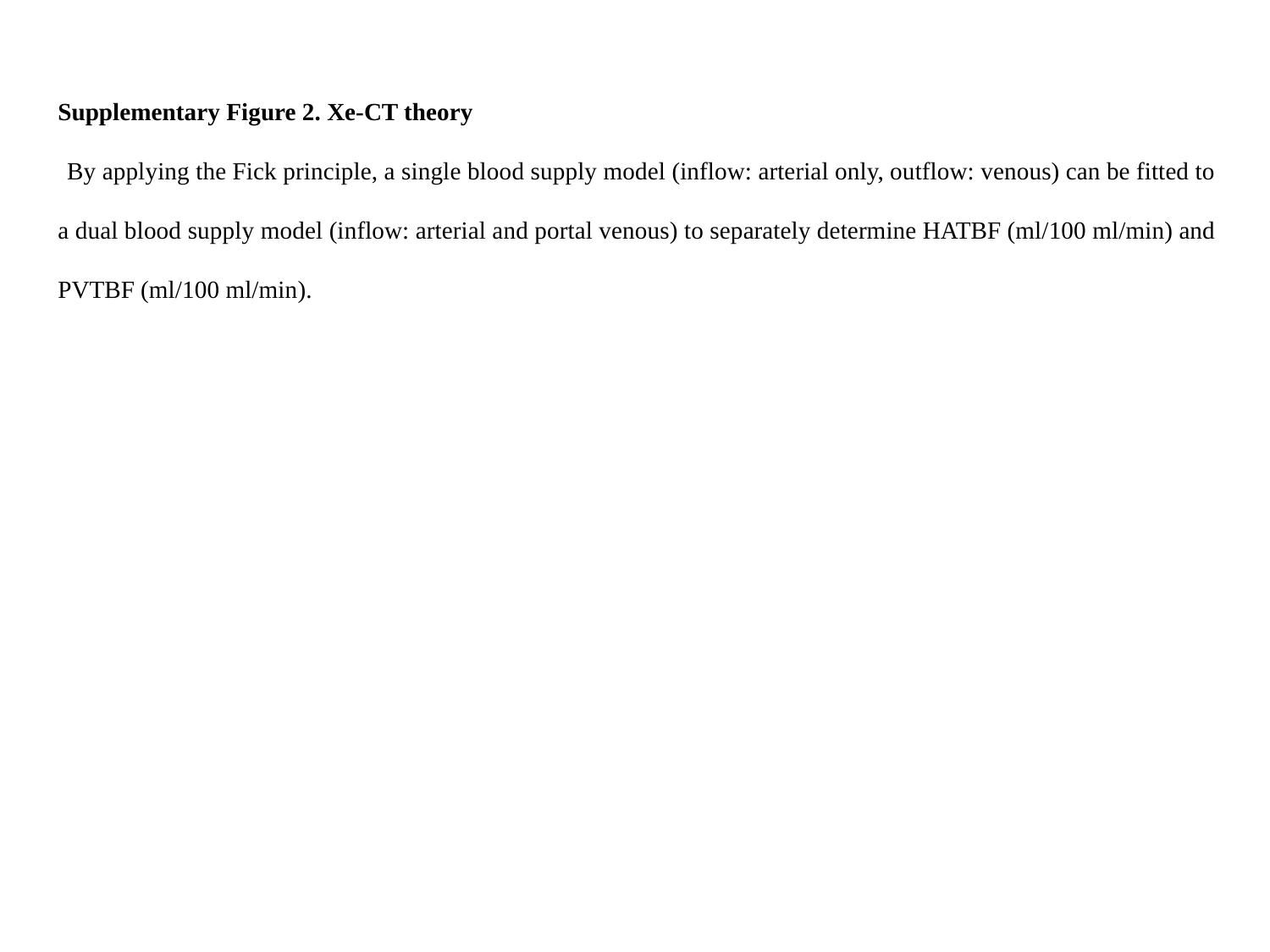

Supplementary Figure 2. Xe-CT theory
By applying the Fick principle, a single blood supply model (inflow: arterial only, outflow: venous) can be fitted to a dual blood supply model (inflow: arterial and portal venous) to separately determine HATBF (ml/100 ml/min) and PVTBF (ml/100 ml/min).
