# Supplementary material for: Effects of endoscopic injection sclerotherapy for esophagogastric varices on portal hemodynamics and liver function
Source: BMC Gastroenterol. 2022 Jul 21;22:350. doi: 10.1186/s12876-022-02422-7 (PMC9306194; doi:10.1186/s12876-022-02422-7)
Supplement: Supplementary file 3 — Additional file 3. Supplementary Figure 3. Xe-CT protocol The wash-in and wash-out periods were of 4 min. The entire liver was CT-scanned at 1-min intervals at four levels, including the porta hepatis (nine scans in total, including the baseline scan). [file 12876_2022_2422_MOESM3_ESM.pptx]

## Slide 1
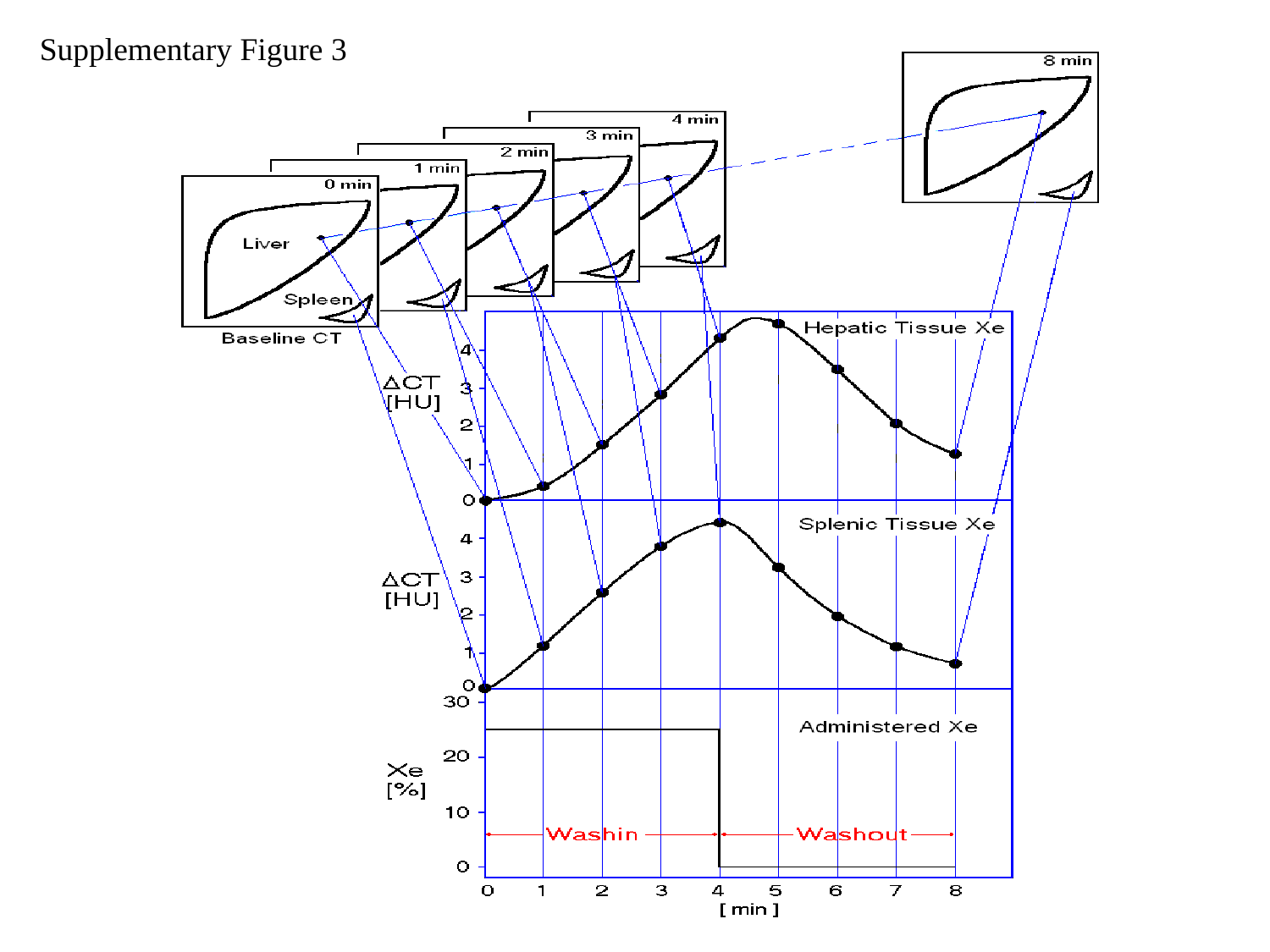

Supplementary Figure 3

## Slide 2
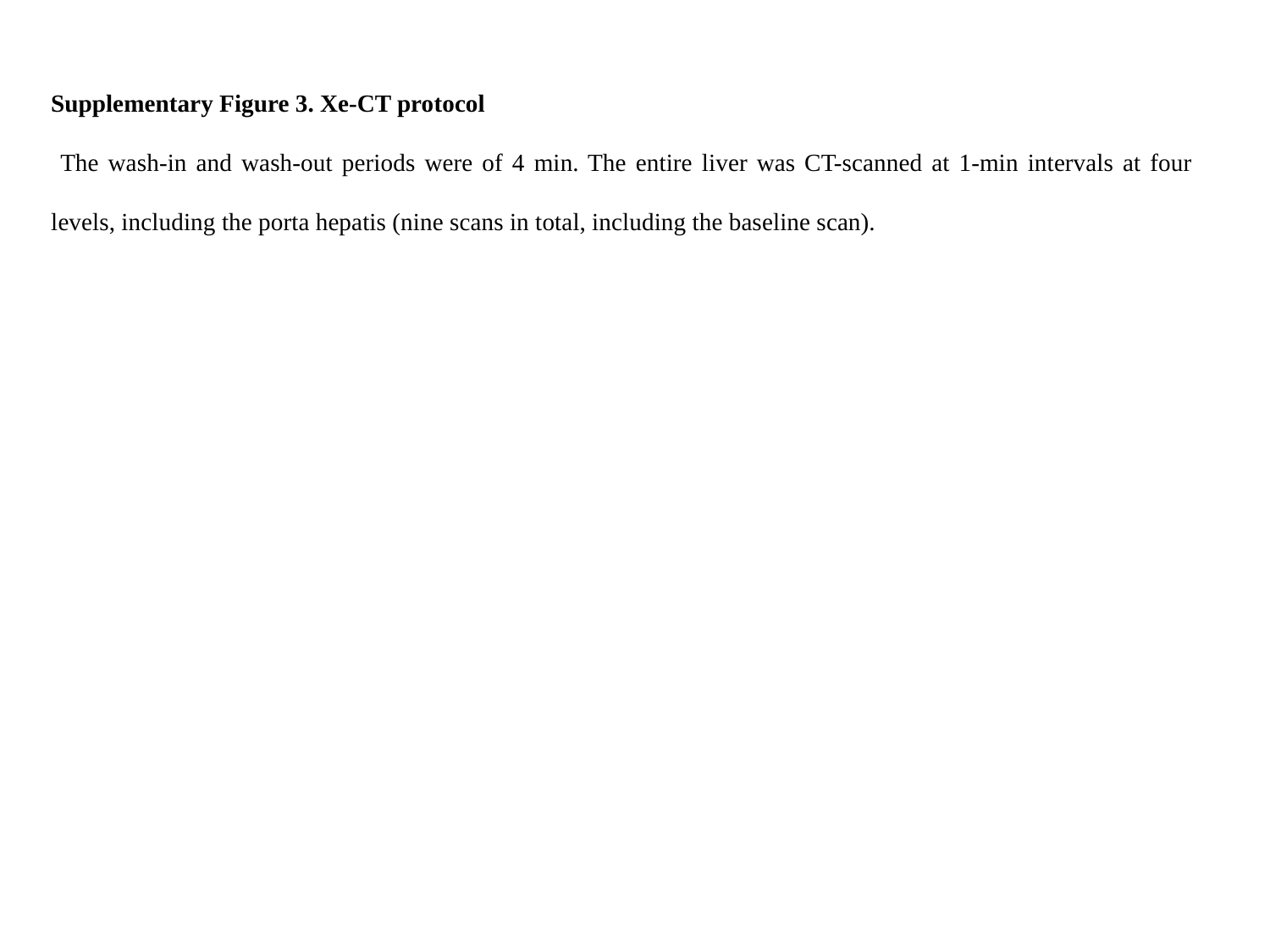

Supplementary Figure 3. Xe-CT protocol
The wash-in and wash-out periods were of 4 min. The entire liver was CT-scanned at 1-min intervals at four levels, including the porta hepatis (nine scans in total, including the baseline scan).
