# Supplementary material for: Effects of endoscopic injection sclerotherapy for esophagogastric varices on portal hemodynamics and liver function
Source: BMC Gastroenterol. 2022 Jul 21;22:350. doi: 10.1186/s12876-022-02422-7 (PMC9306194; doi:10.1186/s12876-022-02422-7)
Supplement: Supplementary file 4 — Additional file 4 Supplementary Figure 4. Endoscopic findings of esophageal varices before and after EIS (A) Before EIS (F2 esophageal varices), (B) After EIS (White cord). EIS has been performed until the varices were eradicated. [file 12876_2022_2422_MOESM4_ESM.pptx]

## Slide 1
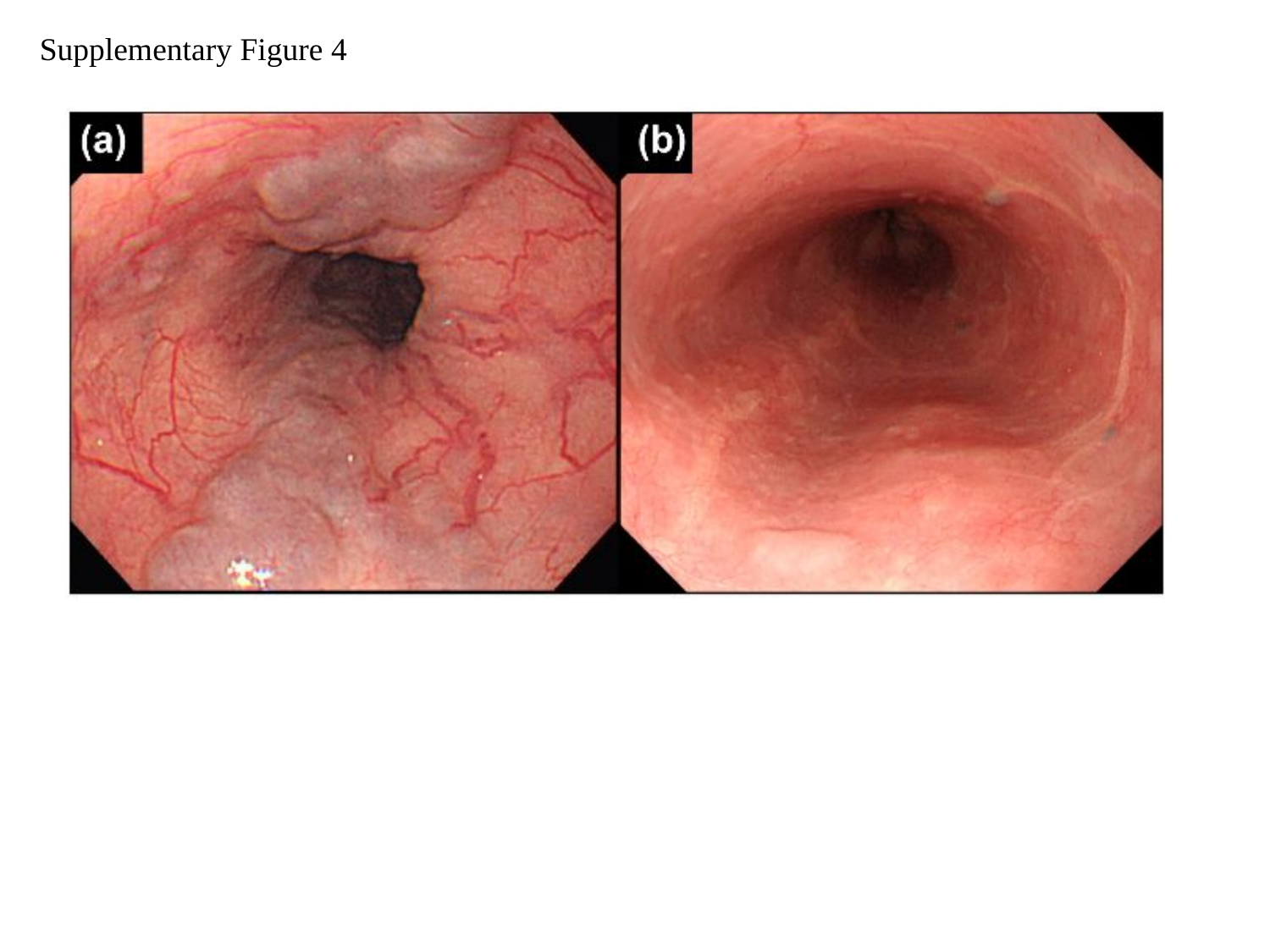

Supplementary Figure 4

## Slide 2
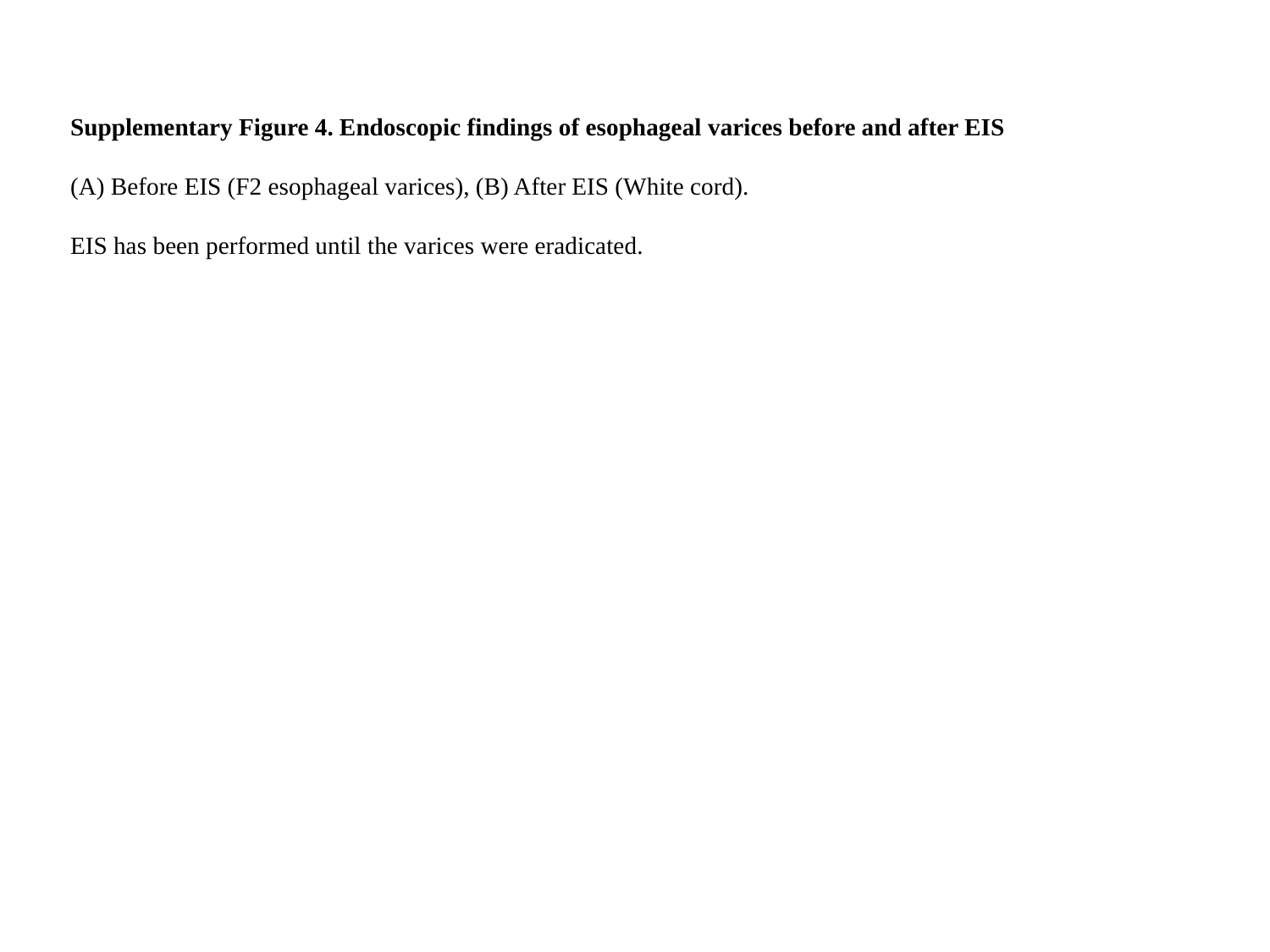

Supplementary Figure 4. Endoscopic findings of esophageal varices before and after EIS
(A) Before EIS (F2 esophageal varices), (B) After EIS (White cord).
EIS has been performed until the varices were eradicated.
